# Superfluous Medical Literature

**Published:** 1908-05-02

**Authors:** 


### Superfluous Medical Literature.

The growth of medical science may be compared to the spread of a large town. It takes place at nearly every part of the circumference; it goes on at an ever increasing rate; it requires, before a definite accretion of finished structures can result, the use of a good deal of scaffolding: for the verbal and written discussion which rages round new ideas may fairly be called part of the scaffolding of the edifice of scientific knowledge. Now modern medicine grows as quickly as well-nigh any other department of contemporary learning, while on the other hand those who practise it have none too much time to meet and talk. For the necessary interchange of their ideas they cannot do without the aid of periodical literature. At present there are certainly no signs of any shortage in the supply. The main subject matter of this literature consists, of course, of findings which have not yet received text-book recognition; and although it is naturally a distinction to add worthily to their number, yet considerable responsibility rests on those who try to do so. For one thing, so very much has been done in the past. Even in the smallest subjects, industrious workers confess that the field of literature is no longer surveyable. Those, therefore, who contemplate adding to it should at least endeavour to find out, by reference to compilations of which the *Index Medicus* was an excellent example, whether or not they can effectively supplement the work of others. They may be quite sure that their notion will *not* be in the category of things "as yet unseized by Germans," for there is nothing new under the sun, and the most riotous originality may find some sort of anticipation in the records of the past. But anyone with the clue of a really original observation which is correct in essentials, can, if he has a little literary faculty and takes the necessary trouble, thread his way through the galleries of the world's medical literature, gathering partial corroboration here and a valuable hint there, until possibly the conception he finally arrives at transcends that of any previous individual worker. Beginners who rush into print with some fragmentary reflection or other should remember the whole of Faraday's advice—"Work, finish, publish."

There is a larger class, however, more hardened offenders because often of some experience and standing in the profession, who have a bad habit of publishing at regular intervals a page or two of solemn, turgid circumlocution. It is to be feared that their sole motive is an interested one; perhaps not the vulgar desire *digito monstrari* (for their papers are often exceedingly moderate and noncommittal in tone), but too probably a prudent care for keeping their name under general notice. Such people obtain a little vogue, but sooner or later their effusions come to resemble those Disraeli commented on so incisively—they produce rather less practical effect than chalking the walls. The modern rage for new books on every conceivable subject makes authors of a good many more. Scarcely a week passes now without announcements in the lay papers of some medical volume with an appeal to the laity. The best works of the sort certainly do good by informing responsible persons and helping to dispel the vast lay ignorance of the theory of medicine. But a fair number seem rather to give dilettantists of various kinds something to discuss, and hypochondriacs something to worry about. In the medical Utopia, priority and frequency of assertion should count for much less than they do now, and the three classes of writers indicated above darken counsel no more.

